# Identifying Patients With Undiagnosed Haemophagocytic Lymphohistiocytosis: Is Ferritin a Useful Screening Tool?

**DOI:** 10.7759/cureus.93916

**Published:** 2025-10-06

**Authors:** Ben Macleod, Elizabeth Peake

**Affiliations:** 1 Haematology, Newcastle Hospitals NHS Foundation Trust, Newcastle, GBR; 2 General Internal Medicine, Queen Elizabeth Hospital, Gateshead, GBR

**Keywords:** haemophagocytic lymphohistiocytosis, hscore, hyperferritinaemia, screening tool, undiagnosed cases

## Abstract

Background

Haemophagocytic lymphohistiocytosis (HLH) is an uncommon hyperinflammatory syndrome characterised by excessive cytotoxic T-cell and histiocyte activity, which can progress to cytokine storm and multi-organ failure. Left untreated, it has a high mortality, but is often diagnosed late or not at all.

Aim

This study aimed to identify patients with undiagnosed secondary HLH at the Newcastle Hospitals NHS Foundation Trust in Newcastle, England, and assess the usefulness of ferritin in developing a screening tool for early recognition of high-risk patients.

Methods

A retrospective analysis of all patients with serum ferritin of >5,000 ug/L was performed. Two established scoring systems for HLH were applied. Clinical features and laboratory values were evaluated, and statistical analysis was applied. Patients’ ages ranged from 19 to 90 years old.

Results

Of 120 patients included in the final analysis, 14 had an HScore of ≥169 (93% sensitive and 86% specific for HLH), of which six were diagnosed with HLH by clinicians.

Conclusion

Utilising the HScore, eight additional patients would have been diagnosed with HLH. Low awareness of HLH amongst clinicians and/or unavailability of key laboratory values may have prevented diagnosis. Ferritin may be a useful component of a screening tool for HLH, especially combined with other key features (fever and cytopaenias). The presence of fever and ferritin of >5,000 ug/L was 100% sensitive and 68.3% specific for sHLH in our study, potentially forming a base from which a screening tool or algorithm could be developed for clinical use.

## Introduction

Haemophagocytic lymphohistiocytosis (HLH) is a hyperinflammatory condition caused by over-activation and prolonged survival of cytotoxic T cells and histiocytes (tissue macrophages and monocyte-derived dendritic cells). Left untreated, it can progress rapidly to cytokine storm and multi-organ failure. It is underdiagnosed and carries a high mortality [1].

HLH is classically divided into primary or familial (pHLH) and secondary or acquired (sHLH) forms. However, recent studies suggest HLH exists on a spectrum, whereby patients have different genetic predispositions requiring a larger or smaller trigger to reach the threshold for cytokine storm. In pHLH, inherited genetic defects generally cause reduced normal T lymphocyte and natural killer (NK) cell activity, leading to overproliferation of abnormal immune cells. Patients require only a trivial trigger to progress to cytokine storm, thus presenting much earlier (generally in infancy and early childhood). Patients with monoallelic or somatic mutations need a larger trigger and would be traditionally classified as having sHLH [2]. In this latter group, HLH often occurs alongside haematological malignancies and rheumatological and immunological conditions, and has been linked to viral infections. sHLH combined with an autoimmune condition is termed macrophage activation syndrome (MAS) [3].

The all-cause mortality of sHLH is 41% [4]. Diagnosis is challenging due to the overlap of HLH symptoms with more common conditions, and specific laboratory tests are unavailable. Historically, diagnostic criteria were developed and validated for pHLH; there are no agreed-upon diagnostic criteria for sHLH. Hyperferritinaemia is a key laboratory feature that may be useful both as a component of screening criteria and for monitoring disease activity and treatment response [5].

In children, a ferritin level of >10,000 ug/L has demonstrated 90% sensitivity and 96% specificity for HLH [1]. There is a wider range of conditions associated with extreme hyperferritinaemia in adults, given their higher baseline iron stores and prevalence of iron overload, liver disease and inflammatory conditions [6]. This is especially relevant for heavily transfused patients.

Other key features of HLH include cytopaenia of multiple cell lineages, hypertriglyceridaemia, hypofibrinogenaemia, hepatosplenomegaly and liver dysfunction. Haemophagocytosis is not required for a diagnosis. Soluble interleukin-2 receptor levels are a promising marker with certain thresholds providing good sensitivity and specificity; however, long processing times limit clinical usefulness [7].

Best practice guidance was released in the UK in 2024, which aimed to improve the detection of HLH and standardise investigations and treatment [8]. Early diagnosis is critical to ensure prompt treatment and prevent progression, and underlying conditions associated with sHLH should be managed as effectively as possible. Intravenous (IV) steroids such as dexamethasone or methylprednisolone are given first-line. Patient outcomes have been shown to improve with treatments such as anakinra [1], and this was recommended for off-label use as the preferred second-line agent to treat HLH in all age groups by NHS England in 2021, instead of or in combination with IV immunoglobulin [9]. Etoposide and ciclosporin can also be used under the supervision of a specialist [8].

This study aimed to identify patients with undiagnosed sHLH at the Newcastle Hospitals NHS Foundation Trust in Newcastle, England, and assess the usefulness of ferritin in developing a screening tool for sHLH, in order to help prompt further testing and application of existing HLH diagnostic criteria, and aid early recognition of high-risk patients.

## Materials and methods

Patient identification

A retrospective analysis was undertaken of patients with a serum ferritin of >5,000 ug/L in the Freeman and Royal Victoria Hospitals between 01/01/2017 and 01/03/2019. This threshold was picked in part due to the hospitals’ internal laboratory critical alert levels: all levels above 5,000 ug/L are logged by the biochemistry department and, if deemed clinically critical, are phoned through to clinicians at the time of result. Recent research has looked into higher cutoff values of ferritin; a historical ferritin cutoff of >500 ug/L was based on paediatric data. A 2020 study by Naymagon et al. concluded that historical threshold levels were too low to be clinically relevant in the adult population [10]. Their study population looked at 1,055 patients across a 10-year period and included adult inpatients with a serum ferritin of >5,000 ug/L. A 2015 research by Saeed et al. in critical care patients suggested that a higher cutoff value of ferritin may improve diagnosis of secondary HLH, with their suggested optimal level being 3,951 ug/L [11]. Adding to this, a 2025 study by Dziedzic et al. examined the characteristics of 21 patients diagnosed with sHLH via adapted HLH-09 criteria over 11 years and found that none of them had a serum ferritin of <5,632 (range: 5,632-30,342, mean: 14,305) [12].

Patients were identified via biochemistry records. In our hospitals, all patients who trigger a critical result have these details logged and stored. Biochemistry staff assisted in obtaining the individual records for all serum ferritin results of >5,000 ug/L in the time period defined above. Where individual patients had multiple ferritin levels of >5,000 ug/L, the clinical presentation corresponding to the peak value was taken. Patients <18 years old and those without data available were excluded. The assay’s upper limit was 16,500 ug/L.

Data was compiled from online records, including admission, referral, discharge and clinic documents, radiography reports and laboratory results. Patient diagnoses listed are those documented by their clinicians.

Diagnosis of HLH

Two main diagnostic tools (Table 1) exist for sHLH: the HScore [13] and the HLH-04 criteria [14]. Research by Debaugnies et al. showed the HScore outperforms the HLH-04 criteria, better controlling for confounding factors that may cause hyperferritinaemia and other features associated with HLH [15]. It also does not require external laboratory tests, which delay diagnosis. Therefore, we used the HScore as our primary diagnostic tool. When applying either scoring system, our researchers were not blinded to the underlying clinical diagnoses. As both scoring systems use objective measures rather than clinical judgement, we did not expect that the lack of blinding would introduce any significant bias.

**Table 1:** HLH scoring systems HLH: haemophagocytic lymphohistiocytosis, AST: aspartate aminotransferase, ALT: alanine transaminase, CD: cluster of differentiation *Human immunodeficiency virus positive or receiving long-term immunosuppressive therapy (i.e., glucocorticoids, cyclosporine and azathioprine) **Scoring for ALT was an alteration made in this study to the original HScore, as most patients had no AST results, whereas ALT was available for all patients ***Meeting of the criteria for either hypertriglyceridaemia or hypofibrinogenaemia awards one point maximum

	Revised HLH-04 criteria	HScore (assigned score in brackets)	
Target population	Secondary HLH	Secondary HLH	
Clinical features			
Fever	Yes	≤38.4 (0)	
			38.4-39.4 (33)	
		No		
	≥39.4 (49)		
			Hepatomegaly	-	Neither (0)	
	Either hepatomegaly or splenomegaly (23)				
Splenomegaly		Yes/no			
	Both hepatomegaly and splenomegaly (38)		
			Immunosuppression*	-	No (0)	
Yes (18)				
Laboratory criteria			
Ferritin (ug/L)	≥500	<2,000 (0)	
		2,000-6,000 (35)	
		>6,000 (50)	
Cytopaenia in ≥2 cell lineages	Two or more of the following: haemoglobin ≤ 90g/L, platelets ≤ 100×10^9^/L, neutrophils ≤ 1×10^9^/L	No/one lineage (0)	
		Two lineages (24)	
		Three lineages (34)	
Hypertriglyceridaemia (mmol/L)	≥3	<1.5 (0)	
		1.5-4 (44)	
		>4 (64)	
Hypofibrinogenaemia (g/L)	≤1.5	≥2.5 (0)	
		<2.5 (30)	
Liver function tests	-	AST < 30 or ALT < 100 (0)	
		AST ≥ 30 or ALT ≥ 100 (19)**	
Haemophagocytosis	Present in bone marrow, spleen or lymph nodes	No (0)	
		Yes (35)	
Natural killer cell activity	Low or absent according to local laboratory reference	-	
Soluble CD25 (Il-2 receptor)	≥2,400 U/mL	-	
Fulfilment of criteria	≥5/8***	Produces a probability outcome - scores > 169 are 93% sensitive and 86% specific for HLH	

Statistical analysis

Population 95% confidence intervals for the mean peak serum ferritin of patients scoring ≥169 on the HScore and of patients scoring ≥5/8 HLH-04 criteria were calculated and rounded to the nearest whole number. Comparison of patient characteristics with HScore > 169 versus HScore < 169 was conducted using the chi-squared test of independence for categorical variables, and the non-parametric Mann-Whitney U test for continuous variables. Two-tailed p-values were considered statistically significant at <0.05. All statistical calculations were performed using freely available tools from Statology© 2025 (statology.org).

## Results

Between 01/01/2017 and 01/03/2019, there were 324 instances of serum ferritin levels > 5,000 ug/L, corresponding to 143 patients. Two patients were excluded due to a lack of data, and 21 were <18 years old. Overall, 120 patients underwent further analysis.

Of the 120 patients, 14 scored ≥169 on the HScore (93% sensitive and 86% specific for HLH [12]) (Table 2). The mean peak serum ferritin was 14,706 ug/L (95% confidence interval: 12,930-16,482). Six of the 14 patients were diagnosed with HLH by clinicians. Three did not qualify as having HLH when using the revised HLH-04 criteria. Conversely, the revised HLH-04 criteria suggested 17 patients had HLH, of which six were diagnosed by their clinicians. Their mean peak serum ferritin was 12,864 ug/L (95% confidence interval: 10,668-15,060). The HScore is thought to be more specific, which may explain this discrepancy between the two scoring systems [15].

**Table 2:** Patients scoring ≥169 of HScore criteria sHLH: secondary haemophagocytic lymphohistiocytosis, ESRD: end-stage renal disease, AKI: acute kidney injury, ALI: acute lung injury, CLL: chronic lymphocytic leukaemia, CMV: cytomegalovirus, HLH: haemophagocytic lymphohistiocytosis, DIC: disseminated intravascular coagulation, ALL: acute lymphocytic leukaemia, AML: acute myeloid leukaemia, GVHD: graft versus host disease, SLE: systemic lupus erythematosus, HSV: herpes simplex virus, APML: acute promyelocytic myeloid leukaemia, EBV: Epstein-Barr virus *Patients diagnosed with HLH by their clinicians

Condition(s) documented	Peak serum ferritin level (ug/L)	HScore	HScore probability of sHLH (%)
ESRD, AKI, ALI, renal transplant	9,107	174	54
Lung transplant, sepsis	>16,500	174	54
CLL, CMV	5,150	177	54
HLH*, Sweet’s syndrome	>16,500	177	54
Massive haemorrhage, DIC	>16,500	181	70
Myeloma	14,349	193	80
HLH*, ALL	>16,500	194	80
ALL, sepsis	>16,500	202	88
AML, GVHD	>16,500	205	88
HLH*, SLE, HSV	12,273	227	96
APML, multi-organ failure	>16,500	241	99
HLH*, bilateral lung transplant	>16,500	267	>99
HLH*, SLE, CMV	>16,500	291	>99
HLH*, EBV	>16,500	321	>99

Further analysis of individual criteria is given in Table 3. Our data also showed that the mean peak serum ferritin level was 58.8% greater in the cohort with an HScore ≥ 169. Mortality figures relating to ferritin levels are included in the Appendices.

**Table 3:** Comparison of patient characteristics based on HScore AST: aspartate aminotransferase, ALT: alanine transaminase †Calculated using the Mann-Whitney U test *Calculated using chi-squared test

	HScore < 169	HScore ≥ 169	p-value (significant if <0.05) (test statistic)
Number of patients	106	14	-
Mean peak serum ferritin level (ug/L)	9,260	14,706	0.00008^†^ (-3.95719)
Patients with haemoglobin < 90g/L	38 (35.8%)	12 (85.7%)	0.000375* (12.6515)
Patients with platelets < 100×10^9^/L	44 (41.5%)	12 (85.7%)	0.001833* (9.7093)
Patients with neutrophils <1×10^9^/L	12 (11.3%)	8 (57.1%)	0.000015* (18.6954)
Patients with a fever	37 (34.9%)	12 (85.7%)	0.000278* (13.214)
Patients with hepatomegaly	11 (10.4%)	7 (50%)	0.000095* (15.2275)
Patients with splenomegaly	18 (17.0%)	10 (71.4%)	<0.00001* (20.4939)
Patients with triglycerides >1.5 mmol/L	6 (5.7%)	10 (71.4%)	<0.00001* (46.2907)
Patients with fibrinogen < 2.5g/L	10 (9.4%)	7 (50%)	0.000043* (16.7361)
Patients with haemophagocytosis recorded	1 (0.9%)	3 (21.4%)	0.00006* (16.1056)
Patients with immunosuppression	31 (29.2%)	12 (85.7%)	0.000035* (17.1505)
Patients with transaminase derangement (AST > 30/ALT > 100)	47 (44.3%)	10 (71.4%)	0.05644* (3.639)

Combinations of values and clinical features from our data, including different ferritin values, were analysed. The presence of fever and ferritin > 5,000 ug/L was 100% sensitive and 68.3% specific for sHLH. Ferritin > 7,000 ug/L with fever gave 82.4% sensitivity and 81.6% specificity. Ferritin > 5,000 ug/L combined with platelets <100×10^9^/L gave 94.1% sensitivity and 59.6% specificity, whilst ferritin > 7,000 ug/L gave only 76.5% sensitivity and 72.7% specificity.

## Discussion

Over 26 months, 143 patients had hyperferritinaemia > 5,000 ug/L in Newcastle Hospitals NHS Foundation Trust, England. In a Swiss single-centre study, 20/42 patients with a serum ferritin > 5,000 ug/L fulfilled the requirements for HLH using a diagnostic algorithm, and 10 patients had a >80% HScore probability [16]. The authors concluded that the HScore reflected a higher specificity and that hyperferritinaemia was strongly associated with HLH in patients with haematological or oncological malignancies.

Whilst HLH is underdiagnosed, it remains rare even in patients displaying severe hyperferritinaemia. Crook and Walker found 41/53,815 ferritin results to be >10,000 ug/L [17]. None were associated with HLH/MAS.

In a Vancouver study by Wormsbecker et al., 83 patients had a serum ferritin > 3,000 ug/L [6]. HLH only caused six (7%) cases, but caused 6/8 (75%) of those with a ferritin > 20,000 ug/L. Similar results were seen in a London study by Thorne et al., where only 7/155 (5%) patients with a serum ferritin > 4,000 ug were diagnosed with HLH [18]. Notably, patients with HLH had the highest median peak ferritin (19,138 ug/L). The authors postulated that HLH may be underdiagnosed due to NK cell activity and soluble cluster of differentiation (CD)25 levels being unavailable.

A multicentre study by Schram et al. found that 19/113 (17%) patients with serum ferritin > 50,000 ug/L had HLH, concluding that there was no value above which ferritin was specific for HLH/MAS [19].

A Chinese series analysed 174 patients with HLH and found that >95% had hyperferritinaemia, high lactate dehydrogenase (LDH), fever and hypoalbuminaemia [20]. Moreover, 92 (52.9%) had haematological diseases, and 55 (31.6%) had Epstein-Barr virus (EBV)/cytomegalovirus (CMV) infections.

Pyrexia of unknown origin, hyperferritinaemia and cytopaenias should raise suspicion of HLH. In a two-year study of 1,329 patients with a serum ferritin > 500 ug/L, 28 (2.1%) were diagnosed with HLH [21]. Hyperferritinaemia was attributed to malignancy in 373 (28.1%) and infection in 343 (25.8%). Thrombocytopaenia and/or multiple lineage cytopaenias were associated with higher odds of having hyperferritinaemia due to HLH. The authors postulated that the combination of serum ferritin > 2,600 ug/L and platelets < 100×10^9^/L could be used as screening criteria for adults at risk of HLH.

Recent guidelines published in the UK offer helpful guidance for clinicians in terms of diagnosing HLH and suggest checking for the presence of fever, hyperferritinaemia and falling counts (cytopaenias) as a screen for severely unwell patients at risk of HLH [8]. However, unlike our study, they do not suggest a specific ferritin value above which HLH is considered much more likely (although they mention that the HScore uses a ferritin value > 2,000 ug/L).

Limitations

One main challenge encountered during data collection was that certain tests were regularly not being requested for patients. Of the 120 patients, 20 had a serum triglyceride result available, 78 had a fibrinogen result available and only five had an aspartate transaminase (AST) result available. These are key components of both diagnostic criteria. Given that one of the stated goals of our study was to assess for any potential missed cases of HLH, it is perhaps expected that some clinicians may not have been aware of HLH as a potential diagnosis in their patients, hence not sending these tests if there was not another clinical reason to do so. There were so few AST results available that, for those without an AST result, we substituted alanine aminotransferase (ALT) as a measure of high transaminase levels. In the original development of the HScore, ALT was initially included, although subsequently dropped in favour of AST due to a less favourable p-value [9]. It is also of note that in their paper, high transaminase levels was a criterion for which the experts did not reach a consensus (i.e., not considered as either “absolutely required” or “not absolutely required”).

The upper limit of the assay for ferritin was 16,500 ug/L, which likely underestimated the true value of some patients’ serum ferritin.

The HScore awards different values corresponding to the severity of fever (≤38.4, 0; 38.4-39.4, 33; ≥39.4, 49). With no access to exact values from bedside charts, all patients with a documented fever scored 33 points.

The above limitations are not expected to have had a significant impact on our study’s primary objectives to assess for potential overlooked cases of secondary HLH and possible components of a screening tool that could be used. HLH may be underdiagnosed due to a lack of awareness or education about the condition, in which case it is understandable why all patients may have not had the required tests for diagnosis sent.

## Conclusions

Our retrospective study showed that, when using the HScore, 14 patients would have been diagnosed with HLH compared to the original six. Patients were potentially missed due to low awareness of HLH amongst clinicians, and triglyceride and fibrinogen results being unavailable.

Ferritin is a quick and cost-effective test that acts as a valuable prognostic indicator in HLH. It may also have value within a screening tool, especially combined with markers including fever and/or thrombocytopaenia. This is relevant to clinical practice, as ferritin is an acute-phase reactant that is often elevated in the presence of any inflammatory process; defining a cutoff ferritin value as part of a screening tool for HLH may help clinicians to more easily distinguish it from other more common inflammatory conditions. Even so, the nonspecific nature of ferritin will always somewhat limit its specificity as a marker in sHLH, and it should be taken in the context of the patient’s presentation and used in combination with established HLH diagnostic criteria. The presence of fever and ferritin > 5,000 ug/L was 100% sensitive and 68.3% specific for sHLH in our study, potentially offering the best utility as a basic screening tool. Further research into sHLH could evaluate the usefulness of combining different ferritin values with the presence of both fever and thrombocytopaenia, perhaps in addition to other common laboratory values, in forming a screening tool.

## Appendices

Table 4 presents mortality figures relating to ferritin levels.

**Table 4:** Patients’ mortality based on peak serum ferritin range

Peak serum ferritin range (ug/L)	Number of patients	Mortality (% of patients who died within 3 months of ferritin measurement)
5,000-6,000	25	24%
6,001-7,000	20	20%
7,001-8,000	11	64%
8,001-9,000	7	43%
9,001-10,000	10	30%
10,001-11,000	4	25%
11,001-12,000	3	0%
12,001-13,000	6	17%
13,001-14,000	6	33%
14,001-15,000	2	50%
15,001-16,000	3	33%
16,000-16,500	1	0%
>16,500	22	50%
